# Conduction Aphasia in a Case of Left Cortical Veins and Left Lateral Sinus Thrombosis Due to Multiple Risk Factors: A Case Report and Review of the Literature

**DOI:** 10.3390/life16060960

**Published:** 2026-06-06

**Authors:** Georgiana Munteanu, Silviana Nina Jianu, Răzvan Bertici, Nicoleta Iacob, Traian Flavius Dan, Dragoș Cătălin Jianu

**Affiliations:** 1 First Division of Neurology, Department of Neurosciences, Victor Babes University of Medicine and Pharmacy, E. Murgu Sq., No. 2, 300041 Timisoara, Romania; munteanu.georgiana@umft.ro (G.M.); traian.dan@umft.ro (T.F.D.); jianu.dragos@umft.ro (D.C.J.); 2 Centre for Cognitive Research in Neuropsychiatric Pathology (NeuroPsy-Cog), Department of Neurosciences, Victor Babes University of Medicine and Pharmacy, 156 L. Rebreanu Ave., 300736 Timisoara, Romania; 3 First Department of Neurology, Pius Brînzeu Clinical Emergency County Hospital, 156 L. Rebreanu Ave., 300736 Timisoara, Romania; 4 Department of Ophthalmology, Dr. Victor Popescu Military Emergency Hospital, 7 G. Lazar Ave, 300080 Timisoara, Romania; silvianajianu@yahoo.com; 5 Department of Multidetector Computed Tomography and Magnetic Resonance Imaging, Scanexpert, 300627 Timisoara, Romania; nicoiacob@yahoo.co.uk

**Keywords:** lateral sinus thrombosis, aphasia, conduction aphasia, left temporal venous infarction, head computed tomography, magnetic resonance imaging, MR venography

## Abstract

Aphasia is a complex neurological syndrome that includes a multitude of signs and symptoms that describe a patient’s inability to use language (understanding and producing spoken and/or written language) after it has already been acquired, which is caused by cerebral lesions situated in the dominant (left) cerebral hemisphere in right-handed people. Aphasia has a prevalence of 25–30% in acute ischemic stroke (especially in arterial infarcts). In patients who suffered cerebral venous and dural sinuses thrombosis (CVST), aphasia has been noticed in almost 20% of cases, its presence being considered a negative predictive factor. We report the case of a 22-year-old right-handed woman with obesity and active smoking (10 cigarettes/day), undergoing treatment with oral contraceptives who presented to the Emergency Department with an intense headache, resistant to usual analgesic treatment, accompanied by language disorders onset within 24 h. The neurological examination was normal, except for language assessment, which revealed the severe impairment of the repetition domain (she was unable to repeat simple words), and difficulty in naming objects with some hesitations and mild comprehension difficulties (especially in complex orders). She underwent neuroimaging examinations at admission. Native Head Computed Tomography revealed spontaneous hyperdensity (parenchymatous hematoma) in the left temporal lobe. Cranial magnetic resonance imaging (MRI) confirmed venous infarction in the left temporal area and a hypointense signal on MRI T2*SW (susceptibility-weighted) in the region of the left lateral sinus and left jugular vein bulb, which confirmed the thrombosis at this level. Associated cortical vein thrombosis was diagnosed on indirect radiological grounds, since hemorrhagic transformation obscured the direct visualization of the adjacent cortical veins. MR venography was not performed at that time, but instead at the 1-month follow-up, MR venography confirmed the chronic, partial thrombosis of the left lateral sinus and left jugular vein bulb. Laboratory data demonstrated an elevated D-dimer and the presence of homozygosity for MTHFR C677T and PAI-1 4G/4G. Anticoagulation in the form of low-molecular-weight heparin was immediately started, followed by chronic treatment with oral anticoagulant (apixaban) and folic acid. The headaches resolved within three days, and her neurological examination was almost normal: the repetition continued being altered for complex phrases. We did not observe any left lateral sinus thrombosis recurrence, or other extra-cerebral embolic events (deep vein thrombosis or pulmonary embolism) during the follow-up year. The immediate anticoagulation since the admission resulted in a favorable outcome. Taking into consideration our interest in monitoring patients with aphasia secondary to CVST, we also analyzed data from the literature regarding the incidence of conduction aphasia and other aphasic syndromes in this CVST. Due to the limited number of articles identified in the last 21 years (2005–2026) in the literature, we concluded that conduction aphasia is an extremely rare clinical presentation in this kind of pathology and further studies should be conducted in order to identify significant statistical data.

## 1. Introduction

Aphasia is a complex neurological syndrome that encompasses a multitude of signs and symptoms that define the inability of a patient to use language (understanding, producing spoken and written language, correlating symbols) after it already has been acquired. Aphasia is the consequence of cerebral lesions situated in the dominant cerebral hemisphere (the left cerebral hemisphere in individuals who are right-handed). Aphasia is present in nearly one-third of patients who have suffered an acute stroke, especially in arterial infarcts [1]. In patients who suffered cerebral venous and dural sinuses thrombosis, aphasia has been noticed in 19–24% of these cases [2,3], its presence being considered a negative predictive factor.

Conduction aphasia is considered one of the least frequent types of aphasia, which is characterized by the alteration of repetition, while both expression (fluency) and comprehension are relatively conserved. In this type of aphasia, the patient is unable to correctly sequence phonological elements, especially when they must repeat polysyllabic words, sentences or phrases [4]. Carl Wernicke was the first who proposed the theory that conduction aphasia is the consequence of a disconnect between these two language systems in the para-Sylvian dominant hemisphere (Broca’s area in the frontal lobe and Wernicke’s area in the posterior region of the superior temporal gyrus) [5]. In the 20th century Lichteim and Geschwind adopted and expanded the theory of Wernicke, arguing that conduction aphasia is the result of a lesion of the arcuate fasciculus, the white matter fiber tract that bonds those two language centers [6,7]. Since the 21st century, Hickok and Poeppel [8] have developed another interesting model (the dual-stream model), based on functional MRI and diffusion tensor imaging studies which have been used to precisely identify language networks, demonstrating that these are more complex than was thought before. Current aphasia models show that language processing occurs in codependent dual pathways:

- the dorsal stream fronto-parietal pathway (responsible for articulatory and syntactic tasks—fluent speech production) is located in the dominant hemisphere and processes auditory to articulation information, connecting the frontal speech areas and the temporo-parietal junction [9];
- the ventral temporal pathway (responsible for decoding sounds to lexical representation and significance to words) is located in both temporal lobes and processes auditory-to-meaning information [4,10,11,12,13,14].

Contrary to classical hypotheses that correlate conduction aphasia with damage to the arcuate fasciculus (a white matter structure), contemporary neuroimaging and clinical evidence suggest that the syndrome primarily stems from focal gray matter lesions. These lesions are strategically situated within the posterior Sylvian fissure and the parieto-temporal junction, a crucial region of the dorsal stream. This interpretation shifts the understanding of the lesion model from a pathology of connectivity (white matter) to a dysfunction of cortical processing (gray matter) within the language network [15,16].

Short-term memory syndrome is also another theory for repetition loss, which is due to lesions that alter critical areas for the working memory: inferior parietal lobule (supramarginal and angular gyri), inferior frontal cortex, posterior temporal lobe, and their white matter connections (the external capsule) [16].

The present case highlights the rarity of conduction aphasia as a first sign for left lateral sinus thrombosis, which accompanies intense headache in a young obese female patient with multiple risk factors (smoker, treatment with oral contraceptives). We proposed to make a review of the literature regarding the frequency of conduction aphasia in patients with cerebral venous and dural sinuses thrombosis.

## 2. Case Presentation

We present the rare case of a 22-year-old right-handed woman, active smoker (10 cigarettes/day), obese, undergoing treatment with oral contraceptives (estrogen–progestin), who presented in the Emergency Department with intense headache, resistant to usual analgesic treatment, accompanied by language disorders, with onset in the past 24 h.

### 2.1. Clinical Presentation

During the general clinical examination, the patient was found to be obese (BMI = 34 kg/m^2^ SC), with normal blood pressure and heart rate (110/70 mmHg and 67 bpm, respectively), and she was afebrile.

By the time of admission, the patient did not undergo an ophthalmological examination. At the 3 week follow-up visit, the ophthalmological examination revealed a normal visual acuity and normal intraocular pressure with no retinal vascular abnormalities.

The neurological examination was normal without nuchal rigidity except for language assessment, which revealed the severe impairment of the repetition domain: she was unable to repeat even simple words, with difficulty in naming objects, repetitive hesitations (word-finding difficulties, self-corrections, phonemic paraphasias), and minor difficulties in comprehension (especially in complex orders).

After the first 24 h from admission (Table 1), a more complex evaluation of language was performed, using the Western Aphasia Battery (WAB) Test Romanian Version, Aphasia Quotient (AQ).

An assessment of oral production (spontaneous speech) revealed fluent spontaneous speech, frequently altered by hesitations and self-correction attempts. We also noticed deviations at various levels: phonemic, verbal, and syntactic. The phonemic level was severely altered by the presence of numerous phonemic paraphasias, less frequent semantic paraphasias, and even neologisms. Verbal level was also affected, our patient being unable to name different objects or actions: anomia. Regarding the syntactic level, the evaluation revealed preserved grammar, even though the sentences were short and simple. The arthric (sound) level was conserved, with normal articulation.

Assessment of repetition was the most difficult, contrasting with the preserved oral comprehension. We noticed the facility of repeating monosyllabic or dissyllabic words, opposing the repetition of polysyllabic words and sentences, which was always incorrect. The patient tended to paraphrase the sentence rather than repeat it, trying to compensate by repetitive self-corrections (“conduite d’approche”—she says an incorrect form of the target word, then makes several phonological adjustments, phonemic paraphasias, gradually getting closer to the correct pronunciation).

Assessment of comprehension revealed that our patient was understanding simple, active sentences, but during the examination of passive sentence comprehension, we noticed that she was guessing the meaning (relatively preserved comprehension).

Assessment of reading and writing demonstrated good reading comprehension, but with regard to paraphasic oral reading the patient had difficulties in spelling and reading unfamiliar words but correctly read and spelled familiar words. The ability of writing was significantly altered with agraphia (she used capital letters, formulated isolated words, and made frequent mistakes in selecting the letters).

### 2.2. Imaging

Our patient underwent neuroimaging examinations at admission. Native Head Computed Tomography revealed spontaneous hyperdensity (parenchymatous hematoma) in the left temporal lateral cortico—subcortical areas (Figure 1).

CT angiography was performed in the acute phase highlighting the lack of opacification of the left sigmoid and transverse sinuses (Figure 2).

Non-contrast cranial magnetic resonance imaging (MRI) performed after 48 h from the admission confirmed venous infarction in the left temporal lateral area (isointensity and hyperintensity in T2-FLAIR) (Figure 3) and a hypointense signal on MRI T2*SWI (susceptibility-weighted imaging) in the same region (Figure 4).

MRI venography was not performed at that time, but instead at the 1-month follow-up visit, MR venography confirmed the chronic, partial thrombosis of left transverse and sigmoid sinuses and the left jugular bulb (Figure 5).

Cortical veins can be visualized through various imaging techniques such as CT, CT venography, MRI, and MR venography. Digital Subtraction Angiography (DSA) remains the gold standard in providing dynamic information especially in collateral venous drainage [17].

In our case, cortical vein thrombosis is supported by specific indirect signs on native CT and MRI: Venous infarction (hemorrhage) in the left temporal lobe with juxtacortical edema is demonstrated by hyperdensity on a non-enhanced CT scan of the brain (Figure 1), which is achieved through isointensity and hyperintensity on T2-FLAIR native (Figure 3) brain MRI, or through a hypointense signal on T2*SWI (Figure 4) native brain MRI (cortical vein thrombosis could not be directly verified because the cortical veins were obscured by the spontaneous FLAIR signal of the left temporal parenchymal hemorrhage).

Due to the rapid diagnosis and administration of low molecular heparin, the venous infarction was limited to a small area.

### 2.3. Laboratory Data

Taking into consideration the patient’s history (obesity, estrogen–progestin oral contraceptives, smoking), clinical spectrum, and the MRI-MRV images, the genetic profiling of thrombophilia was requested and performed, showing inherited thrombophilia: homozygosity for MTHFR C677T and PAI-1 4G/4G, alleles A1/A2—EPCR.

Laboratory data demonstrated minor microcytic anemia (Hgb = 10.21 g/dL, MCV = 77.2/fL), minor thrombocytosis (439,000/uL), inflammatory syndrome (ESR = 45 mm/h), a high level of LDL cholesterol (126 mg/dL), a minor elevated D-dimer level (379.7 ng/mL), and severe Vitamin D deficiency (5.5 ng/mL). We did not find any type of bacterial or viral infections during laboratory findings (urine summary, urine culture, screening viral markers for CHV, BHV, HIV).

### 2.4. Diagnosis

Our female patient presented with the most specific symptom—intense headache, resistance to oral anti-inflammatories—accompanied by a very rare neurologic syndrome, conduction aphasia, the clinical signs of which were the consequence of the left lateral sinus thrombosis and a secondary venous infarction in the temporal lobe–cortico-subcortical areas from the lateral side (left cortical vein thrombosis). Radiological findings were consistent with venous infarction in the left temporal lateral area and left lateral sinus thrombosis. Associated cortical vein thrombosis was diagnosed on the basis of indirect radiological signs, including the juxtacortical topography of the hemorrhagic lesion, surrounding edema, and susceptibility abnormalities. The etiology of this condition is most likely linked to the cumulative pro-thrombotic effects of the inherited thrombophilia, estrogen–progestin treatment, smoking, and obesity. Fortunately, the promptness in establishing the correct diagnosis provided the correct therapeutic approach, with the patient showing favorable progress.

### 2.5. Management, Evolution and Prognosis

In the acute phase, the patient was treated with low-molecular-weight heparin (LMWH) in a therapeutic dosage (100 anti Factor Xa UI/kg b/12 h) administered subcutaneously in two daily doses (each dose/12 h) for 12 days. She also received osmotic diuretics for 72 h (mannitol) for local cerebral edema, along with hydro-electrolytic rebalancing solutions, antiemetics, painkillers, and vitamin B1, B6, and B9.

The patient’s clinical course was favorable, and she was discharged 10 days following admission with residual repetition difficulties as the only persisting issue.

After the acute phase, to prevent recurrent cerebral dural sinuses and/or cerebral venous thrombosis, and even extracerebral venous thrombosis (deep vein thrombosis, pulmonary embolism), given the association of inherited thrombophilia from estrogen–progestin treatment, smoking, and obesity, we recommended oral anticoagulation with NOAC (apixaban) at least in the first 6–12 months from the acute left lateral sinus thrombosis, and the cessation of tobacco and estro-progestative consumption.

Clinical neurological evaluations and neuroimaging assessments were conducted for one, three, six, and twelve months following patient discharge. Ten days after hospitalization, the patient still exhibited subtle repetition disorders, phonemic paraphasias and anomic phenomena (Table 2). One month later, the patient no longer exhibited symptoms of conduction aphasia or any other symptoms.

Given the patient’s previous ophthalmological history and initial transient visual symptoms, ophthalmological monitoring was considered clinically relevant. She had been diagnosed with intraocular hypertension in 2022 and was classified as being at risk for glaucoma; in 2023, periodic ophthalmological evaluation had also identified a superior arcuate scotoma in the right eye. One month after the acute CVST episode, fundus examination showed bilateral venous turgescence, and computerized perimetry revealed relative ring scotoma and a small bitemporal hemianopic scotoma around the fixation point. However, visual acuity remained preserved, and the patient did not report persistent visual field loss or other visual disturbances (Figure 6). Thus, although ophthalmological monitoring was justified by her history, initial symptoms, and venous congestion, no clinically significant persistent visual impairment was observed.

Imaging was performed at 1, 3, 6, and 12 months, gradually demonstrating a partial recanalization (Figure 7) of the left lateral sinus and left jugular bulb. A small sclerotic area (measuring 2/0.4 cm) with FLAIR hyperintensity and minimal hemosiderin deposits, located in the left temporal cortex, remains evident on the non-contrast and contrast-enhanced cranial MRI examination performed at the 12-month follow-up.

No recurrence of left lateral sinus thrombosis or other extracerebral thrombotic events, such as deep vein thrombosis or pulmonary embolism, were observed during the subsequent 12 months of follow-up. The immediate initiation of anticoagulation upon admission contributed to a favorable clinical outcome. Our patient had a rapidly favorable evolution, without neurological sequelae. No adverse events related to anticoagulant therapy were recorded.

## 3. Materials and Methods

With the purpose of identifying other similar cases of conduction aphasia secondary to CVST, our team conducted an extended literature review using the Clarivate Web of Science database.

The search strategy included studies published between 2005 and 2026 and combined terminology related to cerebral venous thrombosis, with terms associated with aphasia and language dysfunction. The content search query used was TS = ((“cerebral venous thrombosis” OR “cerebral sinus thrombosis” OR “dural sinus thrombosis” OR “cerebral vein thrombosis” OR “lateral sinus thrombosis” OR CVT) AND (aphasia OR “language disorder*” OR “language impairment*” OR “speech disturbance*” OR “speech disorder*”)). Only articles and review papers were included, while retracted publications were excluded from our analysis. Similar search strategies were subsequently applied to PubMed and Elsevier databases in order to ensure comprehensive coverage of the available literature. Records were screened first by title and abstract, followed by full-text assessment when the publication appeared potentially relevant. Duplicate records retrieved from different databases were removed manually.

We used the following eligibility/inclusion criteria for our review:

(1) Presence of language disturbances (aphasia, dysphasia);
(2) Presence of CVST and/or dural sinuses thrombosis diagnosed based on an imaging examination: computed tomography (CT) with computed tomography venography (CTV), magnetic resonance imaging (MRI), magnetic resonance venography (MRV), intra-arterial angiography (Digital Subtraction Angiography—DSA);
(3) Articles and review papers published between 2005 and 2026;
(4) Adult human studies.

## 4. Discussion

Conduction aphasia is an uncommon type of fluent aphasia, independent of its underlying causes such as ischemic stroke, hemorrhagic stroke, cerebral tumors, brain trauma, cerebral infections (virus, bacteria, parasite), neurodegenerative diseases, and multiple sclerosis.

Clinical manifestations of conduction aphasia typically encompass various distinctive disturbances at specific linguistic levels, notably affecting repetition. In terms of spontaneous speech, individuals with conduction aphasia generally exhibit fluent expression; however, occasional hesitations and self-corrections may occur, causing only minimal disruption to the overall fluency [13]. With respect to articulation, patients demonstrate no impairment in articulatory function. Additionally, the alteration of phonemic level in patients with conduction aphasia present frequent phonemic paraphasias and less common semantic paraphasias or neologisms [4,13,18]. At the verbal level, there is evidence of moderate anomic characteristics. At the syntactic level, the grammar is mainly preserved, although the sentences are short and have a simplified syntax. An unusual aspect of this fluent aphasia is that comprehension remains intact, unlike what is typically observed in Wernicke’s aphasia. A hallmark feature of conduction aphasia is the pronounced difficulty in repeating polysyllabic words or sentences. The patient paraphrases the sentence rather than repeating it, frequently engaging in self-corrections and repeated paraphasias: attempts to correct phonemic deformations by successive approximations (“conduit d’approche” a term describing repeated efforts to produce the correct word through gradual refinements) [13,18]. For example, the patient might try to say “television,” but instead produces “te-le... tephelon…telephan…telephone”, demonstrating how they approach the correct word through several attempts.

Our patient fulfilled all the characteristic elements identified during clinical evaluation, as detailed in the case presentation.

In relation to determining the cerebral lesion underlying conduction aphasia, it is established that any lesion which impairs the connection between the dorsal and ventral pathways can result in the manifestation of conduction aphasia. Importantly, our study found that the patient’s minor hemorrhagic lesion resulted in considerable language impairment. This outcome may be attributed to local oedema and the strategic location of the lesion in the cortico-subcortical left temporal area (cortical vein thrombosis).

Regarding the risk factors associated with cerebral venous thrombosis, several inherited and acquired conditions have been implicated, although CVST is often multifactorial. Classical inherited thrombophilias, such as factor V Leiden mutation and the prothrombin G20210A variant, have been more consistently associated with venous thrombotic risk. By contrast, the role of MTHFR C677T remains controversial. Although MTHFR C677T homozygosity may contribute to elevated homocysteine levels, current evidence does not support the consideration of this polymorphism alone as a high-risk inherited thrombophilia or an independent direct cause of venous thrombosis. Therefore, in our patient, the presence of MTHFR C677T homozygosity should be interpreted cautiously and within the broader prothrombotic context. The development of CVST was most likely multifactorial, related to the association of estrogen–progestin treatment, active smoking, obesity, and a possible contribution of altered homocysteine metabolism rather than to MTHFR C677T homozygosity alone [3,19,20,21,22,23,24,25,26].

To identify similar cases, we conducted an extended literature search, as described above and summarized in the PRISMA-style flow diagram. The target of the data retrieval was a narrative review of the existing literature. The Web of Science search identified 50 potentially relevant records, while additional searches in PubMed and Elsevier did not identify any further unique publications, only duplicate records. After title and abstract screening, five records were excluded because they were not related to aphasia or cerebral venous thrombosis. Among the 45 reports sought for retrieval, two older self-publications were excluded in order to focus the review on external international data. The remaining 43 reports were assessed for eligibility by full-text assessment where possible. A further 11 reports related to CVST were excluded because they did not contain data on aphasia in the context of cerebral venous thrombosis. In total, 32 publications were included in the narrative qualitative review, consisting of 11 cohort studies or case series and 21 case reports. The most important finding of this review was that no eligible publication described conduction aphasia associated with CVST, supporting the rarity of the present case (Figure 8).

Among the larger cohort studies and case series identified in the literature, language impairment emerged as a relatively frequent neurological manifestation in patients with CVST. In a cohort of 220 patients, language deficits were reported in approximately 42% of cases, particularly in association with hemorrhagic lesions associated with cerebral venous sinus thrombosis [27]. However, other studies reported apparently contrasting findings, with aphasia being more frequently associated with non-Behçet disease CVST [28] and with CVST cases without hemorrhagic lesions [29]. These discrepancies likely reflect the heterogeneity of patient populations, lesion localization, imaging findings, and reporting methodologies across studies.

A multicenter analysis including 624 patients reported aphasia in 119 individuals [30], while a more recent cohort of 147 patients demonstrated that aphasia, without the specification of a subtype, was associated with poorer clinical outcomes [31]. Additional studies further emphasized the prognostic implications of language dysfunction, as aphasia was associated with a higher risk of early neurological deterioration and decreased Glasgow Coma Scale scores in a cohort of 138 patients [32], while another study of 118 patients found that aphasia at presentation predicted the occurrence of decompressive post-surgical seizures [33]. Aphasia was also shown to negatively influence long-term functional and socioeconomic outcomes, with one cohort of 303 patients demonstrating increased employment failure among patients presenting with aphasia [34].

Considerable heterogeneity was also observed in studies examining pregnancy- and puerperium-related CVST. Some reports suggested that aphasia was relatively uncommon in obstetric CVST populations [35], whereas a case series focused on early pregnancy-associated CVST identified aphasia, without further subtype specification, in five out of seven patients [36]. These contrasting findings may similarly reflect differences in the cohort size, timing of presentation, thrombosis localization, and study design.

Case reports demonstrated substantial heterogeneity regarding the aphasic syndromes associated with CVST. Among the 21 case reports, 15 described a specific aphasia subtype, whereas six used only general or non-specific terminology such as aphasia, language disturbance, or speech disturbance.

Hemorrhagic lesions secondary to CVST were frequently associated with severe global aphasia [37,38], non-fluent aphasia [39,40], and sensory aphasia [41]. In one report, an initial anomic aphasia evolved toward a more pronounced expressive aphasia during the clinical course [40]. Other hemorrhagic cases described aphasia without clearly specifying the subtype [42,43], while another report documented “speech disturbance” associated with cytotoxic edema in the setting of COVID-19-related CVST [44].

Ischemic lesions secondary to CVST cases similarly demonstrated a broad spectrum of aphasic manifestations. Non-fluent aphasia was among the most frequently reported presentations [45,46,47]. Global aphasia was also observed in several ischemic CVST cases [48,49]. Fluent aphasia syndromes were less commonly reported and included Wernicke aphasia [50], transient fluent aphasia [51], and isolated anomic aphasia [52]. Some reports described aphasia without further characterization of the language deficit [53,54]. Several cases occurred in specific clinical contexts, including puerperium-associated CVST [47,54], adenocarcinoma-associated hypercoagulability [51], and pediatric infectious complications secondary to Streptococcus anginosus abscesses [55]. Therapeutic approaches varied considerably, with one early case reporting successful treatment using recombinant tissue plasminogen activator (rt-PA) [49].

Several atypical or particularly illustrative clinical scenarios were also identified. One report described CVST mimicking limbic encephalitis because of the predominance of sensory aphasia, emphasizing the diagnostic challenges posed by unusual neurocognitive presentations [56]. Another report documented expressive aphasia associated with vaccine-induced thrombotic thrombocytopenia following ChAdOx1 nCoV-19 vaccination [57]. A summary of the identified cases is provided in Table 3.

A study we conducted in 2022 regarding aphasic syndromes in CVST revealed that non-fluent aphasia and global aphasia were the most prevalent syndromes identified in studies over the past decade [2]. In contrast, fluent aphasia was observed to be exceedingly uncommon, even in cases of left lateral sinus thrombosis, even though this dural sinus represents one of the most frequent sites of thrombosis (38–86%) [2,58,59,60]. The infrequency of this condition may explain the lack of reported cases of conduction aphasia over the past two decades.

After reviewing numerous articles related to CVST and aphasia, we concluded a few hypotheses:

- language deficits were reported in a larger percentage of cases, particularly in association with hemorrhagic lesions secondary to CVST.
- aphasia, without specification of subtype, was associated with poorer clinical outcomes, being associated with a higher risk of early neurological deterioration and decreased Glasgow Coma Scale scores.
- aphasic syndromes are usually identified in patients with venous infarcts secondary to concomitant thrombosis of left lateral sinus and left cortical temporal veins (fluent aphasias), nearly followed by the thrombosis of superior sagittal sinus thrombosis with propagation into tributary fronto-parietal cortical veins (non-fluent aphasias) [2,13].
- the presence of hemorrhagic lesions secondary to CVST were frequently associated with severe global aphasia, non-fluent aphasia, and sensory aphasia.
- non-fluent aphasia and global aphasia were the most prevalent syndromes identified in studies mentioned over the past two decades.

Nevertheless, in the absence of precise scientific data reported in medical literature, and in the presence of inconsistent information, it is almost impossible to extract statistically significant data. What we can conclude is that conduction aphasia was not mentioned in any other cases found in the revised papers.

## 5. Limitations

The present study has several limitations. First, MR venography was not performed during the acute phase, being available only at the 1-month follow-up, which limited direct the confirmation of venous sinus involvement at presentation. Second, advanced structural and functional imaging techniques, such as diffusion tensor imaging (DTI), were not available in our institution. DTI could have provided additional information regarding the integrity of the arcuate fasciculus and other tracts belonging to the dorsal language pathway, potentially offering a more precise anatomical explanation for the conduction aphasia observed in our patient.

The literature review also has methodological limitations. The included studies were highly heterogeneous, consisting of cohort studies, case series, and individual case reports with variable reporting of clinical, imaging, and language assessment data. As a result, the available clinical data was limited, and no consistent pattern could be identified regarding specific aphasic syndromes in relation to the different forms or localizations of cerebral venous thrombosis. For the same reason, the methodological quality of each included publication was not formally assessed using standardized tools such as the Newcastle–Ottawa Scale or the Joanna Briggs Institute critical appraisal checklists. Although the search strategy and eligibility criteria were established by our working group before the review was performed, the protocol was not registered in PROSPERO or another systematic review registry.

Finally, the clinical component of this study is limited by its single-case nature. The absence of previously reported similar cases further supports the rarity of conduction aphasia in the context of cerebral venous thrombosis, but it also prevents broad generalizations based on the present observation alone.

## 6. Conclusions

The main purpose of our study was to highlight a unique case of conduction aphasia secondary to left lateral sinus thrombosis and left cortical vein thrombosis in a young female patient. Another purpose of our paper was to bring attention to the importance of managing the clinical spectrum of language disorders and their relationships to other vascular cerebral causes other than arterial occlusions, especially in the presence of specific risk factors. CVST represents a rare but highly heterogenic cause of stroke, especially in young women (peripartum, post-abortion, consumption of estrogen–progestin) with hereditary thrombophilia, obesity, and active smoking. A prompt diagnosis, made in time, radically changes the course of these patients. Due to lack of articles in the medical literature, research in the field of aphasia due to CVST is relatively poor. Further studies might bring into light other uncovered features.

## Figures and Tables

**Figure 1:**
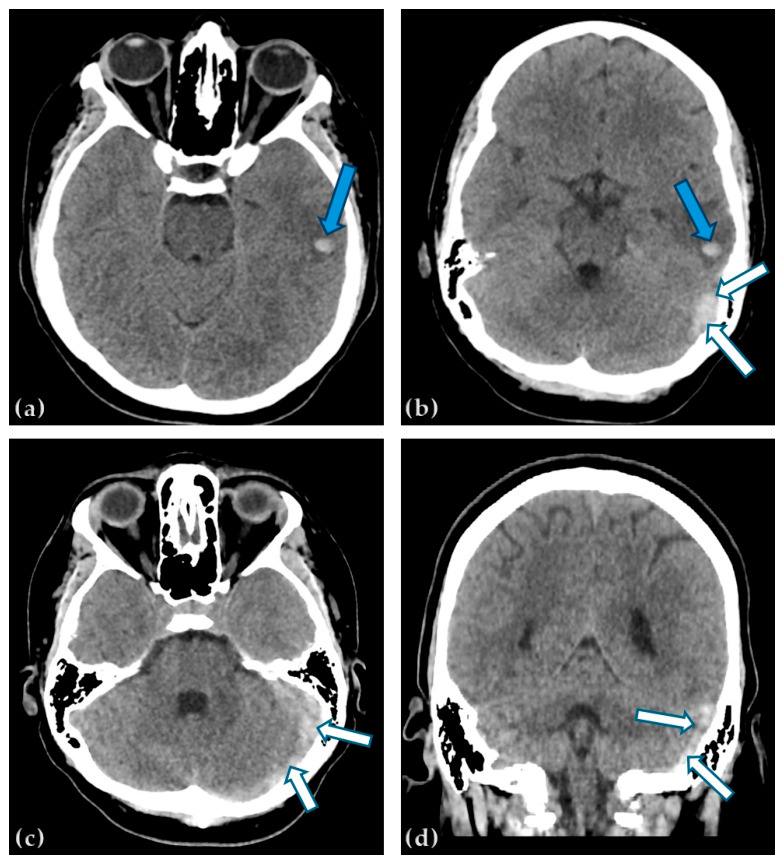
Non-contrast head computed tomography axial (**a**–**c**) and coronal (**d**) revealed spontaneous hyperdensity (parenchymatous hematoma) in the left temporal lateral cortico—subcortical areas (blue arrow) and the hyperdense appearance (acute thrombosis) of the left sigmoid and transverse sinus: visible clot sign (white arrows).

**Figure 2:**
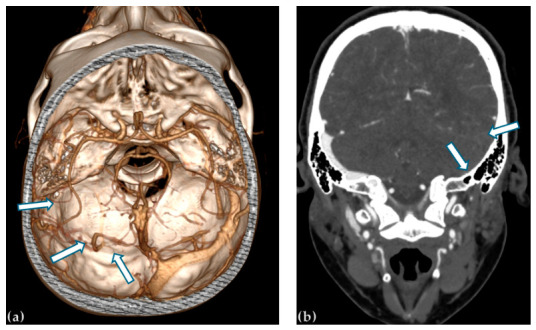
CT angiography performed in the acute phase (**a**,**b**) highlights the lack of opacification of the left sigmoid and transverse sinuses (white arrows). (**a**) VRT; (**b**) MPR coronal.

**Figure 3:**
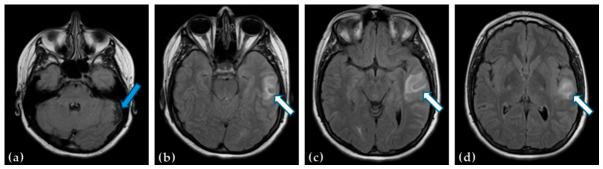
Non-contrast head MRI—T2-FLAIR axial MRI performed in the acute phase shows thrombus ((**a**) hypointensity in the projection of the left lateral sinus—blue arrow) and venous infarction and oedema in the left temporal lateral area ((**b**–**d**) isointensity and hyperintensity—white arrows).

**Figure 4:**
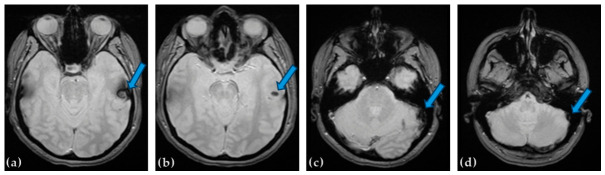
Non-contrast brain MRI performed in the acute phase, axial T2*/SWI sequence. (**a**,**b**) Axial slices through the left temporal region showing hypointense susceptibility changes within the cortico-subcortical venous infarct. (**c**,**d**) Axial slices through the posterior fossa/left lateral sinus projection showing hypointense signal abnormalities along the expected venous drainage pathway (blue arrows).

**Figure 5:**
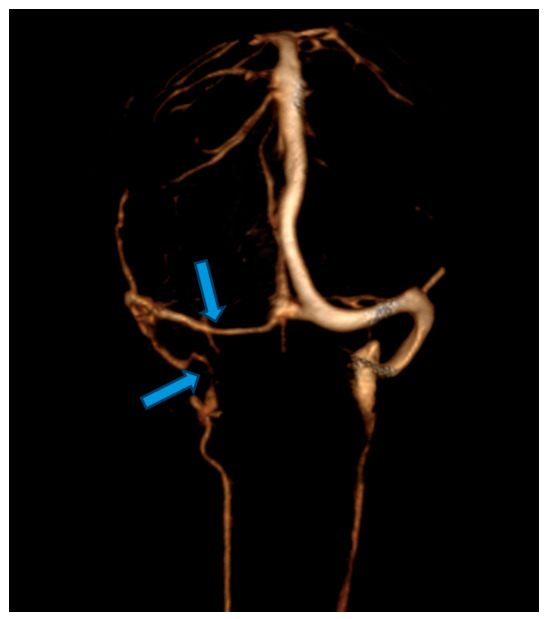
3D-CORONAL VRT (reformatted by CE-MRA) sequence, posterior view; subacute phase shows (1 month) the partial re-permeability of the transverse and sigmoid sinuses with the persistence of partial thrombosis (blue arrows).

**Figure 6:**
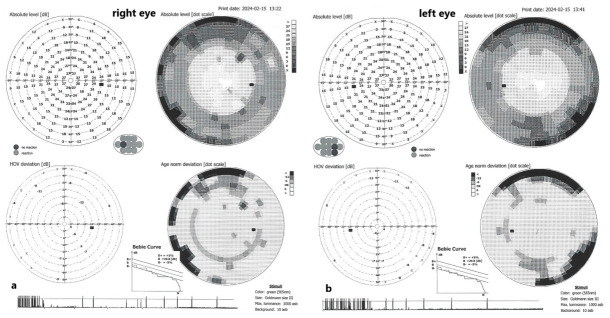
Computerized perimetry ((**a**)—right eye, (**b**)—left eye), relative ring scotoma between 20 and 60 degrees, bitemporal hemianopsic scotoma 5 degrees around fixation point.

**Figure 7:**
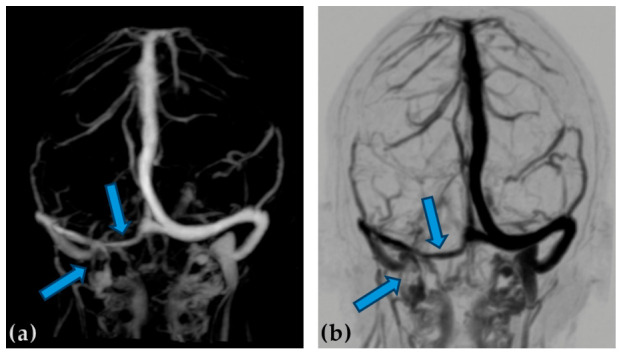
MRI venography (**a**,**b**) at 6 months from the admission revealed the partial recanalization of the left lateral sinus and left jugular bulb (blue arrows).

**Figure 8:**
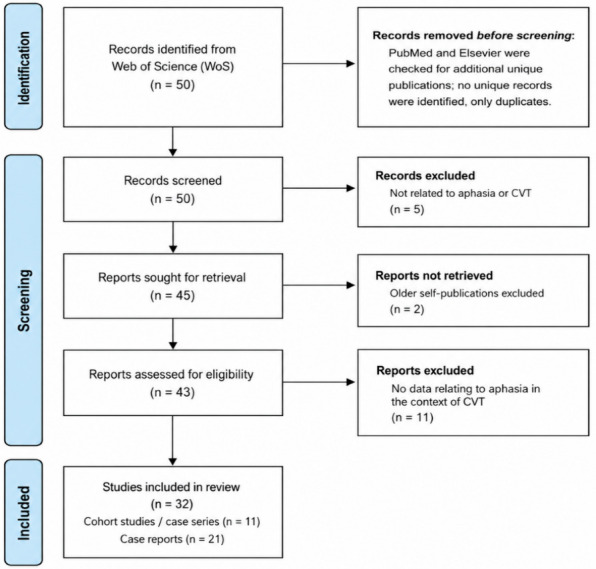
PRISMA-style flow diagram of the literature review process.

**Table 1:** Western Aphasia Battery Test Romanian Version at the first 24 h from admission.

Fluency	Comprehension	Repetition	Naming	Aphasia Quotient (AQ)
13/20	9.1/10	5.1/10	7.5/10	69.4/100

**Table 2:** Western Aphasia Battery Test Romanian Version at 10 days after admission.

Fluency	Comprehension	Repetition	Naming	Aphasia Quotient (AQ)
20/20	10/10	7.4/10	9/10	92.8/100

**Table 3:** Detailed breakdown of identified cases of CVST with aphasia from the literature.

Authors	Year	Aphasia Type	Thrombosis Localization	Parenchymal Lesion
Falavigna et al. [42]	2006	Not specified	Superior sagittal, transverse and sigmoid sinus bilaterally and numerous tortuous cortical veins	Hemorrhagic venous infarction
Misra et al. [49]	2007	Global aphasia	Not specified	Ischemic venous lesion
Zivanović et al. [39]	2011	Broca aphasia	Not specified	Hemorrhagic venous infarction
Guney [41]	2011	Wernicke aphasia	Left transverse and sigmoid sinus and internal jugular vein	Hemorrhagic venous infarction
Kuan [40]	2014	Anomic aphasia	Left transverse and sigmoid sinus and internal jugular vein	Hemorrhagic venous infarction
Pizzi et al. [38]	2016	Global aphasia	Left transverse and sigmoid sinuses	Hemorrhagic venous infarction
Rahme et al. [37]	2019	Global aphasia	Trolard vein	Hemorrhagic venous infarction
Lan et al. [47]	2020	Broca aphasia	Left transverse sinus	Ischemic venous lesion
Yeşilbaş [55]	2020	Not specified	Superior sagittal sinus	Abscess
Ennis et al. [48]	2021	Global aphasia	Superior sagittal and right lateral sinuses	Hemorrhagic venous infarction
Das et al. [45]	2021	Broca aphasia	Superior sagittal and bilateral transverse sinuses.	Ischemic venous lesion
Saito et al. [53]	2021	Not specified	Left transverse sinus and superior sagittal sinuses	Ischemic venous lesion
Bersinger et al. [57]	2021	Broca aphasia	Superior sagittal sinus and cerebral veins	Ischemic venous lesion
Engelmann et al. [51]	2021	Wernicke aphasia	Left transverse sinus	Not specified
Karatsu et al. [43]	2022	Not specified	Sphenoparietal sinus and superficial middle cerebral vein	Hemorrhagic venous infarction
Çakmakci et al. [44]	2023	Not specified	Not specified	Cytotoxic edema
Dias da Costa et al. [52]	2023	Anomic aphasia	Deep venous system	Ischemic venous lesion
Oshima et al. [46]	2023	Broca aphasia	Deep cerebral venous thrombosis	Ischemic venous lesion
Tezuka et al. [56]	2024	Wernicke aphasia	Left sigmoid, transverse, superior sagittal, and straight sinuses	Not specified
Fard et al. [50]	2025	Wernicke aphasia	Left transverse and sigmoid sinuses	Ischemic venous lesion
Castleberry et al. [54]	2025	Not specified	Not specified	Ischemic venous lesion

## Data Availability

The data is not publicly available. Anonymized data may be provided upon request from the principal authors.
